# A Topology Visualization Early Warning Distribution Algorithm for Large-Scale Network Security Incidents

**DOI:** 10.1155/2013/827376

**Published:** 2013-09-26

**Authors:** Hui He, Guotao Fan, Jianwei Ye, Weizhe Zhang

**Affiliations:** Department of Computer Science and Technology, Harbin Institute of Technology, Harbin, Heilongjiang 150001, China

## Abstract

It is of great significance to research the early warning system for large-scale network security incidents. It can improve the network system's emergency response capabilities, alleviate the cyber attacks' damage, and strengthen the system's counterattack ability. A comprehensive early warning system is presented in this paper, which combines active measurement and anomaly detection. The key visualization algorithm and technology of the system are mainly discussed. The large-scale network system's plane visualization is realized based on the divide and conquer thought. First, the topology of the large-scale network is divided into some small-scale networks by the MLkP/CR algorithm. Second, the sub graph plane visualization algorithm is applied to each small-scale network. Finally, the small-scale networks' topologies are combined into a topology based on the automatic distribution algorithm of force analysis. As the algorithm transforms the large-scale network topology plane visualization problem into a series of small-scale network topology plane visualization and distribution problems, it has higher parallelism and is able to handle the display of ultra-large-scale network topology.

## 1. Introduction

With the network's application in various areas of human life, network security draws more and more attention all over the world. Network security problems such as computer virus and hackers' illegal intrusion lead to important information leaks and may even cause the network paralysis. The accidents have caused huge economic losses to various countries and many companies and even endanger the security of the countries and regions. Just in the first half of 2004, nearly 2 million hosts were attacked by major worms like Mydoom, RPC loopholes, and LSASS loopholes in China [1, 2].

The study of early warning system and intrusion detection technology has already carried out in many countries. These systems monitor the illegal intrusion in some important economic, political, and military networks. They play important roles in the protection of network security, the early detection of intrusion, and the control of virus' spread. There is no suitable intrusion detection and early warning system for large-scale network in China at present. In order to support the information system and adapt to the requirement of information warfare, it is necessary to develop the large-scale network intrusion detection and early warning system. It has very important significance to improve the network system's emergency response capabilities, alleviate the cyber attacks' damage, and strengthen the system's counterattack ability.

The large-scale network system's plane visualization is realized based on the divide and conquer thought. First, the topology of the large-scale network is divided into some small-scale networks by the MLkP/CR algorithm [3]. Second, the subgraph plane visualization algorithm is applied to each small-scale network. Finally, the small-scale networks' topologies are combined into a topology based on the automatic distribution algorithm of force analysis. As the algorithm transforms the large-scale network topology plane visualization problem into a series of small-scale network topology plane visualization and distribution problems, it has higher parallelism and is able to handle the display of ultralarge-scale network topology.

## 2. Related Works

Some international research institutions have been engaged in the study of this aspect. In 1999, the Information Assurance Advisory Council (IAAC) conducted a project called Threat Assessment and Early Warning Methodologies for Information Assurance [4, 5]. It mainly develops and evaluates the analysis methods for threat assessment and early warning. The research goal is to prove that quantifiable threat assessment and early warning are feasible, which lays foundation for further application research. The achievements are as follows: (1) to prove the feasibility of threat outline's generation and describe the threat outline from the attacker's motives, intentions, capabilities, and behavioral patterns and (2) to argue the feasibility of indicating and alarming computer attacks from substate stage actor behavior.

The study has several limitations. It focused on network's external threats. The considered attacker type is limited to sub state stage attacker; the state or national agent stage attackers are not considered. Many theories and technologies involved in the project are not mature and still need further research and development. Another related project is Information Warfare Attack Assessment System [4], developed by the International Centre for Security Analysis (ICSA) of British Kingps College London between 1997 and 2000. This project presented the concept of information warfare attack's threat assessment, indication, and warning, as well as the conceptual framework of an open information source decision support system. Its goals are as follows: (1) to evaluate the information warfare threat caused by different attackers, (2) to provide information warfare attack's indication and warning, and (3) to predict the enemy's behavioral path. The above two projects are of great relevance.

In China, researches have been done on attack detection technology and feature information extraction methods of network security strategic early warning system [6, 7]. Overall, only a small number of domestic agencies are working on network security early warning system, and there is no much open technical literature available. And existing intrusion detection systems just simply submit alarm information to administrator by a format of record. Administrator can hardly get the distribution state of current network's abnormalities from the boring records, and it is also not conducive to deal with abnormalities in time. Under this background, this paper proposes a large-scale network security incident early warning system which combines active measurement and anomaly detection, aiming at macro-early-warning for the outbreak of wide range network events based on network topology. And we will focus on the visualization of the early warning system. Through this system the administrator can get the security events' distribution state intuitively in graphical form. 

Therefore, this paper comes up with a plane visualization problem and probes into how to locate large-scale network topology on plane and get good visual effect at the same time.

## 3. Topology Distribution Visualization Technology for Large-Scale Security Incidents

### 3.1. Plane Visualization Algorithm Framework

According to the balanced subproblem thought of the divide and conquer algorithm, the original topology should be decomposed with the following requirements: (1) to decompose the original topology into *k* subtopology with the same scale less than *N*; each subtopology is described as *G*_*i*_(*N*_*i*_, *V*_*i*_), so *N*_*i*_ < *N*; (2) to make the edges in each subtopology as few as possible and keep each subtopology independent as far as possible, so that each subproblem will be independent and at the same time the subtopologies will have good locality; thus the administer can observe a subtopology's information relatively independently; (3) and to ensure that each subtopology is a connected graph, according to the divide and conquer algorithm, as the network topology is an undirected connected graph, so each subgraph is a logical network topology.

As a result, the plane visualization algorithm framework for large-scale network is shown in Algorithm 1. 

The key technologies of the algorithm include core router screening technology, undirected graph segmentation algorithm MLkP/CR, the subgraph internal vertex distribution algorithm, quasi-planarity technology, and the subgraph automatic distribution algorithm based on force analysis. This paper focuses on explaining the core router screening technology and the subgraph automatic distribution algorithm based on force analysis.

The scale of each subgraph is defined as *N*, so the algorithm realizes the plane visualization of undirected graph with *k*∗*N* scale. The algorithm is able to solve the plane visualization problem of undirected graph with any size, but the value of *k* is different, *k* = |*G*_0_ | /*N*. In particular, the problem becomes a small undirected graph plane visualization problem when *k* = 1. The algorithm has high parallelism, and the subgraph plane visualization can be realized in parallel.

### 3.2. Core Router Screening Technology

As the connection relations of core routers constitute the backbone of the network topology, the plane visualization of the topology backbone is the key to the plane visualization of the network graph. Before dividing the network topology, we can find its backbone and reduce its scale through cutting. The topology is defined as *T* = (*V*, *E*), where |*V*| = *n* and |*E*| = *m*. As *T* is connected, so the minimum degree of the vertexes in *T* is 1. Cut down the vertexes with degree 1 in *T*; a new topology *T*′ = (*V*′, *E*′) is got. The vertexes in *T*′  are the vertexes in *T* with the degree more than 2. If the number of the vertexes in *T* with degree 1 is *t*, then |*V*| = *n* − *t* and |*E*| = *m* − *t*. So if *t* changes sharply, the number of the drawn vertexes will be greatly reduced when this method is used iteratively. If *T* is equal to *T*_0_ at the beginning; after several iterations we get *T*_1_, *T*_2_, …, *T*_*n*_ and *D*_1_, *D*_2_, …, *D*_*n*_, where *D*_*k*_ = *T*_*k*−1_ − *T*_*k*_; namely, *D*_*k*_ is constructed by the vertexes with degree 1 and the edges connecting these vertexes in *T*_*k*−1_. If we can draw *T*_*n*_ at this time, then we can draw *T*_0_ reversely. The method is as follows: according to the definition of *D*_*n*_, a vertex *v* in *D*_*n*_ is mapped to a unique vertex *s* in *T*_*n*_; select a position around *s* and draw *v*. After that, connect *s* and *v*. Deal with the vertexes in *D*_*n*_ circularly and get a new topology including all the vertexes and edges in *D*_*n*_ and *T*_*n*_. As *D*_*n*_ + *T*_*n*_ = *T*_*n*−1_, so we can get *T*_0_, namely, *T*, by calling this method iteratively.

### 3.3. Subgraph Macro–Automatic-Distribution Algorithm Based on Force Analysis

**Figure 1:**
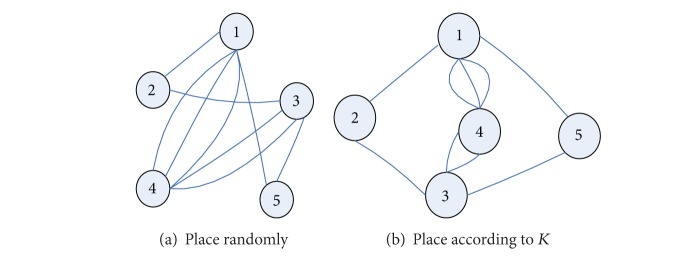
The influence of the subgraphs' positions on cross-edges.

After the plane visualization of each subgraph is realized, put the subgraphs together and connect them with the edges between them. Then a whole topology, namely, the large-scale undirected graph before divided, is got. Therefore, the plane visualization of large-scale undirected graph is realized. For any two subgraphs *G*_*a*_ and *G*_*b*_, their associated value is defined as *K*(*a*, *b*) = ∑*e*(*u*, *v*), *u* ∈ *G*_*a*_∧*v* ∈ *G*_*b*_. If *K*(*a*, *b*) = 0; then the associated value is the minimum and there are no edges between the two subgraphs. If *K*(*a*, *b*) > *K*(*c*, *b*), then the associated value of *G*_*a*_ and *G*_*b*_ is bigger than that of *G*_*c*_ and *G*_*b*_. As *K* is different between different subgraphs, the subgraphs cannot be put together randomly. Their associated values *K* are related to their mutual positions, and the cross of the edges can be reduced by putting the subgraphs with higher *K* together. As shown in Figure 1, there are 5 subgraphs; on putting the subgraphs with higher *K* together, the cross can be reduced and the connect relation between subgraphs can be shown more clearly.

The goal of the distribution algorithm is to make the distance between the subgraphs as even as possible and make the cross edges as few as possible, namely, trying to put the subgraphs with higher *K* together.

For a given undirected connected graph *G*(*V*, *E*), it is made up with m subgraphs {*G*_*m*_, *G*_*m*−1_ ⋯ *G*_1_} and their edges. The distribution space of *G* is defined as *L*_*m*∗*m*_, a matrix with *m*∗*m* scale. For *i*, *j* ∈ {0 ⋯ *m* − 1}, if graph *G*_*k*_ captures the space, then make *L*[*I*, *j*] equal to *k*  (*k* > 1). If *L*[*I*, *j*] is 0, then the space is not captured by any subgraphs. *X*[*i*] presents the start abscissa of the space with row *i* and *Y*[*j*] presents the start ordinate of the space with column *j*. The abscissa range of *L*[*I*, *j*] is (*X*[*i*] ~ *X*[*i*] + *G*_*i*_ · length) and the ordinate range is (*Y*[*i*] ~ *Y*[*i*] + *G*_*i*_ · width).

Now we import the force analysis method to our algorithm. With the above method, the subgraph distribution is transformed into the matrix distribution, and the number of each subgraph indicates the position where it is in the whole graph. Take the distribution matrix as a box, each subgraph waiting for distribution as a quality pellet, and the edges as rubber band. If the value of *K* is different, then the elastic coefficient of the rubber band is different. The rubber band has a free length. If it is pulled, then tension is generated. There is repulsion between any two pellets. Through this physical system, the subgraphs' placing process in the matrix is transformed into the process that the pellets move in the box according to mechanics laws and ultimately achieve balance. The pellets' positions in the box when balance is achieved are the subgraphs' right positions in the matrix.

The physical formula is defined as follows:

(1) tension formula:
  (1) |tension(vi,vj,ek)| ={0,(length(ek)≥distance(vi,vj)),k∗(distance(vi,vj)−length(ek)),
(2) repulsion formula:
  (2) |repulsion(vi,vj)| ={f,(distance(vi,vj)=0),g∗mass(vi)∗mass(v(distance(vi,vj)≠0)j)distance(vi,vj)2.

The pellet's quality is proportional to the subgraph's degree. Compared to the star structure, the greater the center vertex's degree, the heavier its quality and the greater the repulsion, and then the vertexes around it will have more space to distribute. As distribution in the matrix can't be as accurate as that in the physical world, we use a kind of greedy algorithm to distribute the subgraphs. It can avoid the accurate calculation in real physical world and achieve good effect at the same time. Distribute the subgraphs according to the pellets' qualities. As *d* ∝ |*F* | /*m*, the greater the quality is, the less the displacement is [8]. And at the same time, the greater the degree is, the bigger the influence on the graph's distribution is. Therefore, the subgraph with higher quality is distributed preferentially.

Once the distribution of all the subgraphs is decided, their positions are fixed and will not be changed. As a result, when the next subgraph waiting for distribution is *i*, the positions of its previous *i* − 1 subgraphs are already certain and we only need to consider the influence of the previous *i* − 1 subgraphs. Here we use the traversal algorithm. Traverse every position of the matrix when it is put in pellet *i* and find the position where the force is the minimum; it is also the balance and final position of pellet *i*.

Distribution algorithm based on force analysis is shown in Algorithm 2. 

## 4. The Experimental Results and Analysis

### 4.1. The Experiment Results of Core Router Screening

**Figure 2:**
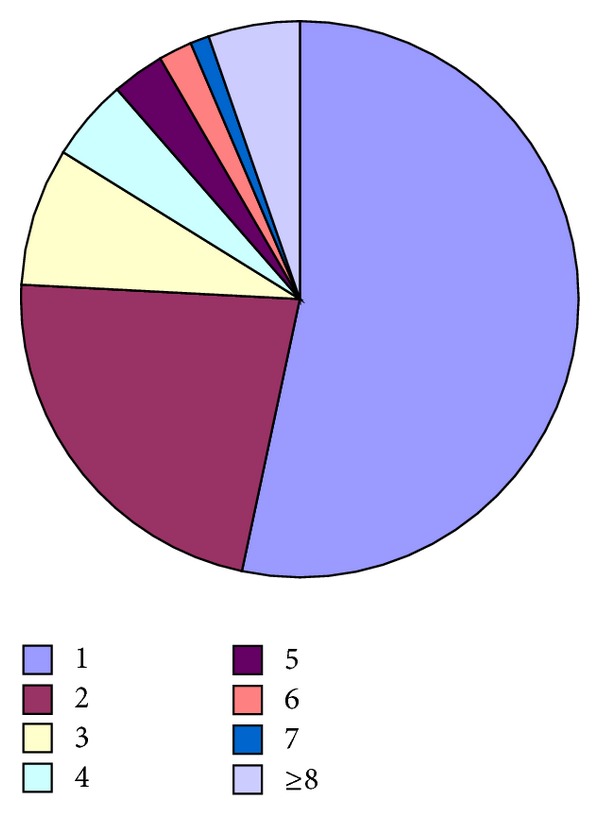
The degree distribution map of all the routers in China.

The degree distribution of all the routers in China is shown in Figure 2. There are 19847 routers in the network of China. Among these routers, 53.25% are routers with 1 degree. The network scale can be reduced by half through cutting off these vertexes, because they are the leaves attached to the topology backbone, and it is easy to add them to the backbone when the backbone is drawn. Finally, the plane visualization is realized.

### 4.2. The Experiment Results of the Distribution Based on Force Analysis

The effect of the distribution based on force analysis is related to the effect of the random distribution; the more the two kinds of distributions are a like, the better the algorithm's randomness is, the greater the dispersion degree of the distribution is, and the more balancedly the vertexes distribute. The experiment results of the two kinds of distributions are shown in Figures 1 and 2. The coordinate is the subgraph's row and column position in the distribution matrix.

Next, we compare the effect of the two kinds of distributions by calculating the distance of the data in Tables 1 and 2 and the distribution algorithms' results.

The distribution distance between two subgraphs is defined as

(3) $$\begin{array}{c} d=\sqrt{{\left(x_{1}-x_{2}\right)}^{2}+{\left(y_{1}-y_{2}\right)}^{2}}. \end{array}$$

The ratio of all the distances' mean absolute deviation and average is defined as *α* and it is calculated by (4). *α* indicates the well-proportioned degree of the subgraphs. The smaller *α* is, the better proportionally the subgraphs are distributed.

(4) $$\begin{array}{c} \alpha =\frac{\left(1/m\right)\sum \left|d_{i}-\overset{-}{d}\right|}{\overset{-}{d}}. \end{array}$$

**Figure 3:**
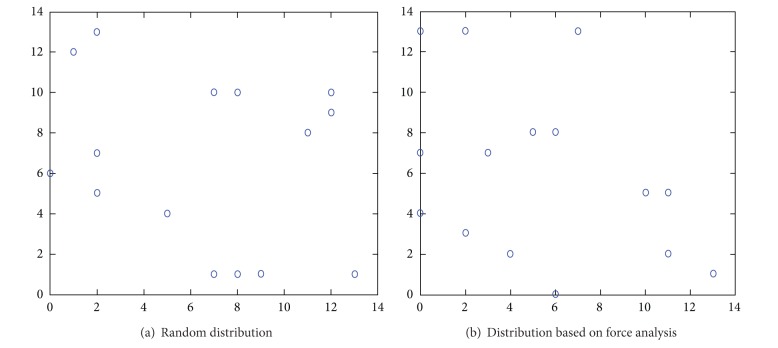
Distribution diagram of the subgraphs.

Table 3 is calculated from Tables 1 and 2. We can see that there is little difference between the two *α*, which means that the effect of the two kinds of distributions is almost the same and the force makes effective use of the distribution space, as shown in Figure 3.

**Figure 4:**
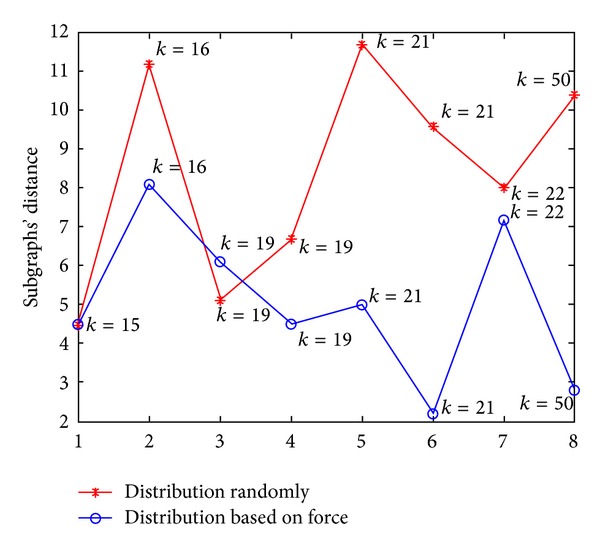
Comparison of the subgraphs' distance when *k* > 15.

The distribution based on force analysis can reflect the relationship and related degree of the subgraphs. Calculate parameter *K* which measures the distance between two subgraphs. From Figure 4 we can see that for the same value of *K*, the subgraphs' distance of the distribution based on force analysis is smaller than that of the random distribution, which says that the distribution based on force analysis reflects the relationship between the subgraphs. As the value of *K* becomes greater, the subgraphs' distance of the distribution based on force analysis presents degressive tendency. It means that our algorithm reflects the related degree of the subgraphs; the bigger the value of *K*, the greater the related degree and the smaller the distance. But the random distribution can't express these characters.

The *α* of the distribution algorithm based on force analysis is almost the same as that of the random distribution algorithm, meaning that the average degrees of the two kinds of distributions are similar. While the subgraphs' distribution is related to the value of *K* in the distribution algorithm based on force analysis, the subgraphs with greater *K* are closer. Therefore, the distribution result of the distribution algorithm based on force analysis is better than that of the random distribution algorithm.

### 4.3. The Experiment Results of the Network Logical Topology in China

**Figure 5:**
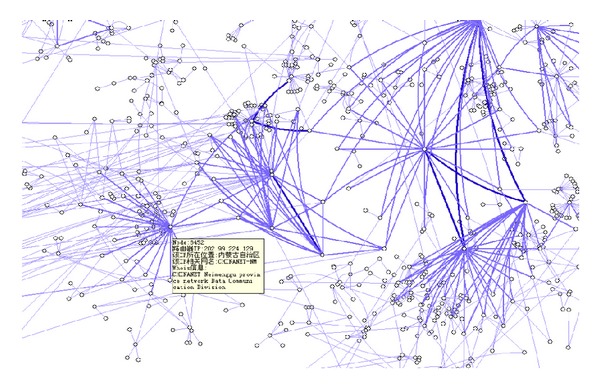
The subgraph display of the network logical topology in China.

**Figure 6:**
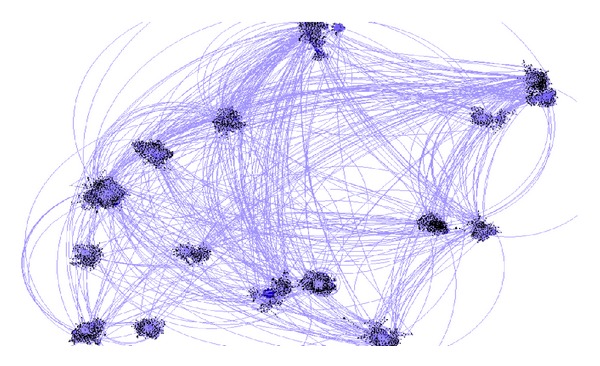
The whole display of the network logical topology in China.

The scale of the network in China is 10^4^ stage. It has 19847 points and 24864 edges. Obviously, it is a large-scale network. The experiment results are shown in Figures 5 and 6.

## 5. Conclusion

This paper proposes a whole framework of the plane visualization algorithm based on the divide and conquer thought, aiming at solving large-scale network plane visualization problems, and it probes into two of the key algorithms and technologies applied in this algorithm. (1) Core router screening technology: we use this technology to cut down the leaf nodes and get the main stem of the graph. It can reduce the scale of the graph and improve the graph's plane visualization efficiency. Experiment has shown that after processed by this technology, the scale of the graph can be halved. (2) Subgraph automatic distribution algorithm based on force analysis: with physicalification ideology, we transform the subgraph distribution problem into physical problem and get a reasonable distribution in the distribution space. Compared with the random distribution, experiments show that our algorithm has its superiority.

## Figures and Tables

**Algorithm 1:**
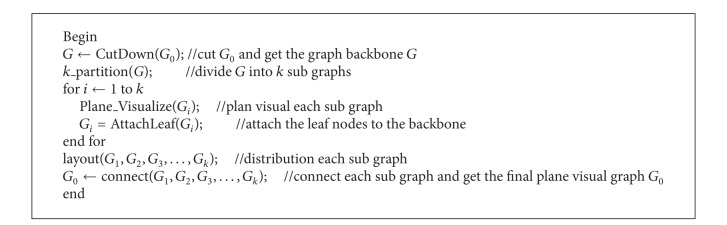
Plane visual algorithm.

**Algorithm 2:**
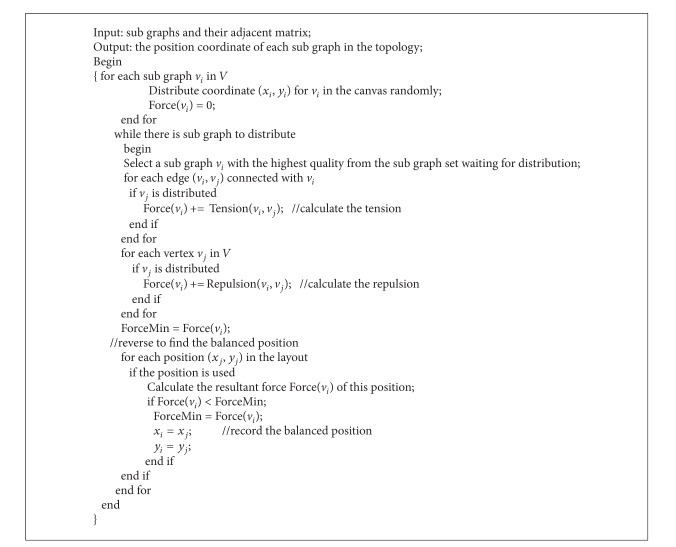


**Table 1:** Results of random distribution.

Cluster no.	Coordinate
1	(12, 10)
2	(8, 1)
3	(0, 6)
4	(1, 12)
5	(13, 1)
6	(7, 1)
7	(11, 8)
8	(12, 9)
9	(2, 5)
10	(9, 1)
11	(8, 10)
12	(2, 13)
13	(2, 7)
14	(5, 4)
15	(7, 10)

**Table 2:** Results of distribution based on force analysis.

Cluster no.	Coordinate
1	(6, 0)
2	(3, 7)
3	(6, 8)
4	(5, 8)
5	(13, 1)
6	(11, 2)
7	(2, 3)
8	(0, 13)
9	(10, 5)
10	(11, 5)
11	(0, 4)
12	(2, 13)
13	(4, 2)
14	(7, 13)
15	(0, 7)

**Table 3:** Comparison of the two *α*.

	Random	Based on force analysis
*α*	0.0021	0.0028

## References

[1] Karypis G, Kumar V (1998). Multilevel k-way partitioning scheme for irregular graphs. *Journal of Parallel and Distributed Computing*.

[2] Jiang Y, Hu M, Fang B, Zhang H (2002). An Internet router level topology automatically discovering system. *Journal of China Institute of Communications*.

[3] Taşdemir K, Merényi E (2009). Exploiting data topology in visualization and clustering of self-organizing maps. *IEEE Transactions on Neural Networks*.

[4] Ankerst M, Breunig MM, Kriegel H, Sander J OPTICS: ordering points to identify the clustering structure.

[5] Krishnamurthy B, Wang J Topology modeling via cluster graphs.

[6] Krishnamurthy B, Wang J On network-aware clustering of web clients.

[7] Peng Z, Grundy E, Laramee RS, Chen G, Croft N (2012). Mesh-driven vector field clustering and visualization: an image-based approach. *IEEE Transactions on Visualization and Computer Graphics*.

[8] Bruckmann F, Gruber F, Cundy N, Schäfer A, Lippert T (2012). Topology of dynamical lattice configurations including results from dynamical overlap fermions. *Physics Letters B*.

